# Knee extension strength measurements should be considered as part of the comprehensive geriatric assessment

**DOI:** 10.1186/s12877-018-0815-2

**Published:** 2018-06-01

**Authors:** Suey S. Y. Yeung, Esmee M. Reijnierse, Marijke C. Trappenburg, Gerard J. Blauw, Carel G. M. Meskers, Andrea B. Maier

**Affiliations:** 1 Department of Human Movement Sciences, @AgeAmsterdam, Faculty of Behavioural and Movement Sciences, Vrije Universiteit Amsterdam, Amsterdam Movement Sciences, Van der Boechorststraat 7, 1081 BT Amsterdam, The Netherlands; 2 Department of Medicine and Aged Care, @AgeMelbourne, The Royal Melbourne Hospital, The University of Melbourne, Melbourne, Australia; 3 Department of Internal Medicine, Section of Gerontology and Geriatrics, VU University Medical Center, Amsterdam, The Netherlands; 4 Department of Internal Medicine, Amstelland Hospital, Amstelveen, The Netherlands; 5 Department of Gerontology and Geriatrics, Leiden University Medical Centre, Leiden, The Netherlands; 6 Department of Geriatrics, Bronovo Hospital, The Hague, The Netherlands; 7 Department of Rehabilitation Medicine, VU University Medical Center, Amsterdam, The Netherlands

**Keywords:** Muscle strength, Geriatric assessment, Aged

## Abstract

**Background:**

Comprehensive geriatric assessment (CGA) generally includes handgrip strength (HGS) as a measure of overall muscle strength that is associated with various health characteristics in geriatric outpatients. Whether this is also true for knee extension strength (KES) is yet unknown. This study aims to compare the associations between health characteristics from the CGA with both HGS and KES in geriatric outpatients.

**Methods:**

Data were retrieved from a cross-sectional study. A total of 163 community-dwelling older adults referred to a geriatric outpatient clinic of a middle-sized teaching hospital were included. Health characteristics included physical, nutritional, social, psychological, diseases, cognitive, and behavioural factors. HGS and KES were assessed three times for each limb and the best performance was used for analysis. Sex-specific z-scores of HGS and KES were used to allow comparison of effect estimates. Associations between health characteristics with standardized HGS and KES were analysed with linear regression adjusted for age, sex and further adjustment for standardized KES (for model of HGS) or standardized HGS (for model of KES).

**Results:**

Physical, nutritional and psychological health characteristics were positively associated with both HGS and KES after adjustment for age and sex, with overall stronger associations with KES compared to HGS. All significant associations with HGS were lost after further adjustment for KES; significant associations with KES remained after further adjustment for HGS, except for nutritional characteristics.

**Conclusions:**

Stronger associations of health characteristics with KES compared to HGS indicate its additional value and therefore inclusion of KES in the CGA is recommended.

## Background

Decline in muscle strength is a well-known consequence of the aging process [1]. Low muscle strength is associated with various health characteristics including lower functional status, dependency in activities of daily living (ADL) and instrumental activities of daily living (IADL) [2, 3], risk of malnutrition [2], poorer cognitive function [4, 5], higher morbidities [2] and mortality [6]. To quantify muscle strength, handgrip strength (HGS), followed by knee extension strength (KES) is the most often used measure in clinical practice. Measuring HGS is simple and grip dynamometers are portable and inexpensive [7] while measuring KES is more technically challenging and not widely accessible in clinical practice [8].

Whether HGS, KES or both should be measured in clinical practice is still controversial. Independent of age and health status, HGS and KES showed low agreement at both population and individual level in five cohorts encompassing healthy young and old individuals, geriatric outpatients and older individuals post hip fracture, suggesting that HGS or KES measurement cannot be used interchangeably [9]. Among community-dwelling older adults, lower HGS and KES of the same individual were independently associated with poorer physical performance [10, 11]; while others demonstrated that physical performance had stronger positive association with KES compared to HGS [12, 13]. Comprehensive geriatric assessment (CGA) is a multi-dimensional, inter-disciplinary diagnostic process for older adults to determine a range of health characteristics (physical, functional, psychological and social), followed by implementation of a co-ordinated plan for treatment and follow up [14]. It raises the question whether health characteristics from the CGA other than physical performance are more associated with HGS or KES and which measurement or both should be used in clinical practice.

The aim of this study is to compare the associations between health characteristics from the CGA with both HGS and KES in geriatric outpatients.

## Methods

### Setting

This cross-sectional study included 299 community-dwelling older adults referred to a geriatric outpatient clinic in a middle-sized teaching hospital (Bronovo Hospital, The Hague, The Netherlands) due to mobility problems for the CGA between March 2011 and January 2012. The CGA included questionnaires and measurements of physical and cognitive function and was performed by trained nurses or medical staff. The study was reviewed and approved by the institutional review board of the Leiden University Medical Center (Leiden, the Netherlands). Individual informed consent was waived since the study was based on regular care. Ethical guidelines were followed in accordance with the Declaration of Helsinki. Of the 299 outpatients, data on KES was available in 163 outpatients due to a protocol amendment in which the measurement of KES was added in a later stage. Therefore, data on 163 geriatric outpatients was used in the present analysis.

### Muscle mass and muscle strength

Direct segmental multi-frequency bioelectrical impedance analysis (BIA; InBody 720; Biospace Co., Ltd., Seoul, Korea) was used to measure body composition. Measures included fat mass percentage (total fat mass divided by total body mass), lean mass percentage (total lean mass divided by total body mass) and appendicular lean mass percentage (sum of lean mass in all four limbs divided by total body mass).

HGS was measured using an isometric hand dynamometer (JAMAR hand dynamometer: Sammons Preston, Inc., Bolingbrook, IL) in an upright standing position with the arms along the side, while holding the dynamometer in one hand. The width of the dynamometer’s handle was adjusted to hand size. Three trials were performed alternately for each hand [15]. Geriatric outpatients were actively encouraged to squeeze with maximal strength. The best performance was used for analysis and expressed in kilograms.

KES was measured in a seated position, with hips and knees in 90 degrees, by a force transducer mounted in a chair (Forcelink Inc., Culemborg, The Netherlands). Outpatients were asked to push with maximal effort against a cuff positioned just above the talocrural joint. Three trials were performed for each leg. The best performance was used for analysis and expressed in Newton meters (Nm).

### Health characteristics

Age and sex were extracted from the medical charts. Health characteristics were grouped into the following domains.

#### Physical

Self-reported fall in the previous 12 months, Short Physical Performance Battery (SPPB) [16] expressed in points, Timed Up and Go test (TUG) [17] in seconds and ADL in points were included. The SPPB comprises the ability to maintain balance in three different standing positions with eyes open, a timed four-meter walk, and a timed sit-to-stand test. Higher SPPB score indicates higher degree of lower extremity functioning [16]. For the TUG test, outpatients were asked to rise from a chair, walk for three meters, then turn and walk back to the chair and sit down. Faster time in the TUG test indicates greater physical mobility [17]. ADL was assessed by four criteria: the need of assistance with bathing, clothing, toilet, or transfers [18].

#### Nutritional

The Short Nutritional Assessment Questionnaire (SNAQ) includes three questions on loss of appetite, unintentional weight loss and the use of sip or tube feeding [19]. The composite SNAQ score is used for analysis to indicate the risk of malnutrition. Body weight and standing height were measured to the nearest 0.1 kg and 0.1 cm respectively and body mass index (BMI) was calculated.

#### Social

Information on care support (receiving either informal or formal care support) and living arrangements (dependent living was defined as living in residential care or assisted living) were self-reported.

#### Psychological

Anxiety and depression was assessed using the Hospital Anxiety and Depression Scale (HADS) [20], which was added in a later stage due to protocol amendment. Higher scores represent more anxiety and depressive symptoms.

#### Diseases

Information regarding the number of diseases and medications was extracted from medical records. Multimorbidity was defined as the presence of two or more diseases, including chronic obstructive pulmonary disease, heart failure, diabetes mellitus, hypertension, malignancy, myocardial infarction, Parkinson disease, (osteo)arthritis, transient ischemic attack, and stroke. Polypharmacy was defined as having five or more medications.

#### Cognitive

Global cognitive function was assessed using the Mini-Mental State Examination (MMSE) [21] and the Montreal Cognitive Assessment (MoCA) [22].

#### Behavioural

Information on current alcohol use (expressed as the number of units of alcohol consumed per week) and current smoking was self-reported.

### Statistical analysis

Continuous variables with a normal distribution were presented as mean (standard deviation (SD)). Variables with a skewed distribution were presented as median (interquartile range (IQR)). Categorical variables were presented as number (n) and percentage (%).

HGS and KES were standardized into sex-specific z-scores to allow comparison of effect estimates. Linear regression analyses were performed to examine the association between different health characteristics (independent variables) and standardized muscle strength measures (dependent variables). Analyses were performed with adjustment for age and sex (model 1). To determine the strongest association of health characteristics with HGS and KES, effect estimates (β) were compared and significance was tested with the formula ((β_1_- β_2_)/ √(SE_1_^2^ + SE_2_^2^)) and *p*-values derived from the standard normal distribution table.

The association between health characteristics and HGS was further adjusted for standardized KES to test independency of HGS (Model 2). The association between health characteristics and KES was further adjusted for standardized HGS to test independency of KES (Model 3). Data were presented as effect estimates (β) and standard error (SE). Results of the linear regression analysis with standardized variables can be interpreted as: βxSD gives the difference between outpatients with the presence of the health characteristics of the muscle strength measures, compared to outpatients without the presence of the health characteristics.

Data were analysed using Statistical Package for the Social Sciences, version 24.0 (SPSS Inc. Chicago, IL, USA). A two-tailed *p*-value of less than 0.05 was considered statistically significant. Visualization of results was performed using GraphPad Prism 5.01.

## Results

### Characteristics of geriatric outpatients

Table 1 shows the characteristics of geriatric outpatients. The mean age was 81.7 years (SD 7.2) and 39.3% were male. Mean BMI was 25.8 kg/m^2^ (SD 4.6) and the prevalence of multimorbidity and polypharmacy were 38.2 and 61.6% respectively.

**Table 1:** Characteristics of geriatric outpatients

	N	Total (*n* = 163)
Age, years	163	81.7 (7.2)
Males, n (%)	163	64 (39.3)
Height, m	159	1.67 (0.10)
Fat mass, %	111	32.5 (9.3)
Lean mass, %	111	63.5 (8.8)
ALM, %	111	28.0 (4.6)
Physical		
Fall in previous 12 months, n (%)	163	114 (69.9)
SPPB score, median [IQR]	158	7 [5–10]
TUG, seconds, median [IQR]	146	16.0 [12.2–21.5]
ADL score, median [IQR]	132	0 [0–0]
Nutritional		
SNAQ score, median [IQR]	154	0 [0–1]
BMI, kg/m^2^	155	25.8 (4.6)
Social		
Care support, n (%)	155	97 (62.6)
Dependent living^a^, n (%)	160	22 (13.8)
Psychological		
HADS depression score, median [IQR]	111	5 [3–9]
HADS anxiety score, median [IQR]	111	6 [4–9]
Diseases		
Multimorbidity^b^, n (%)	157	60 (38.2)
Polypharmacy^c^, n (%)	159	98 (61.6)
	N	Total (n = 163)
Cognitive		
MMSE score, median [IQR]	163	27 [24–29]
MoCA score, median [IQR]	132	23 [19–25]
Behavioural		
Alcohol use, units/week	160	0 [0–7]
Current smoking, n (%)	136	21 (15.4)
Muscle strength		
Handgrip strength, kg	163	
Male		33.0 (5.5)
Female		21.5 (4.9)
Knee extension strength, Nm	163	
Male		111.2 (42.5)
Female		61.6 (21.7)

All values are presented as mean (SD) unless indicated otherwise

*ALM* appendicular lean mass, *SPPB* Short Physical Performance Battery, *IQR* Interquartile range, *TUG* Timed Up & Go, *ADL* Activities of daily living, *SNAQ* Short Nutritional Assessment Questionnaire, *BMI* body mass index, *MMSE* Mini-Mental State Examination, *MoCA* Montreal Cognitive Assessment, *HADS* Hospital Anxiety and Depression Scale

^a^Defined as living in residential care or assisted living

^b^Defined as ≥ 2 diseases

^c^Defined as ≥ 5 medication

### Association between health characteristics with handgrip strength and knee extension strength

**Fig. 1:**
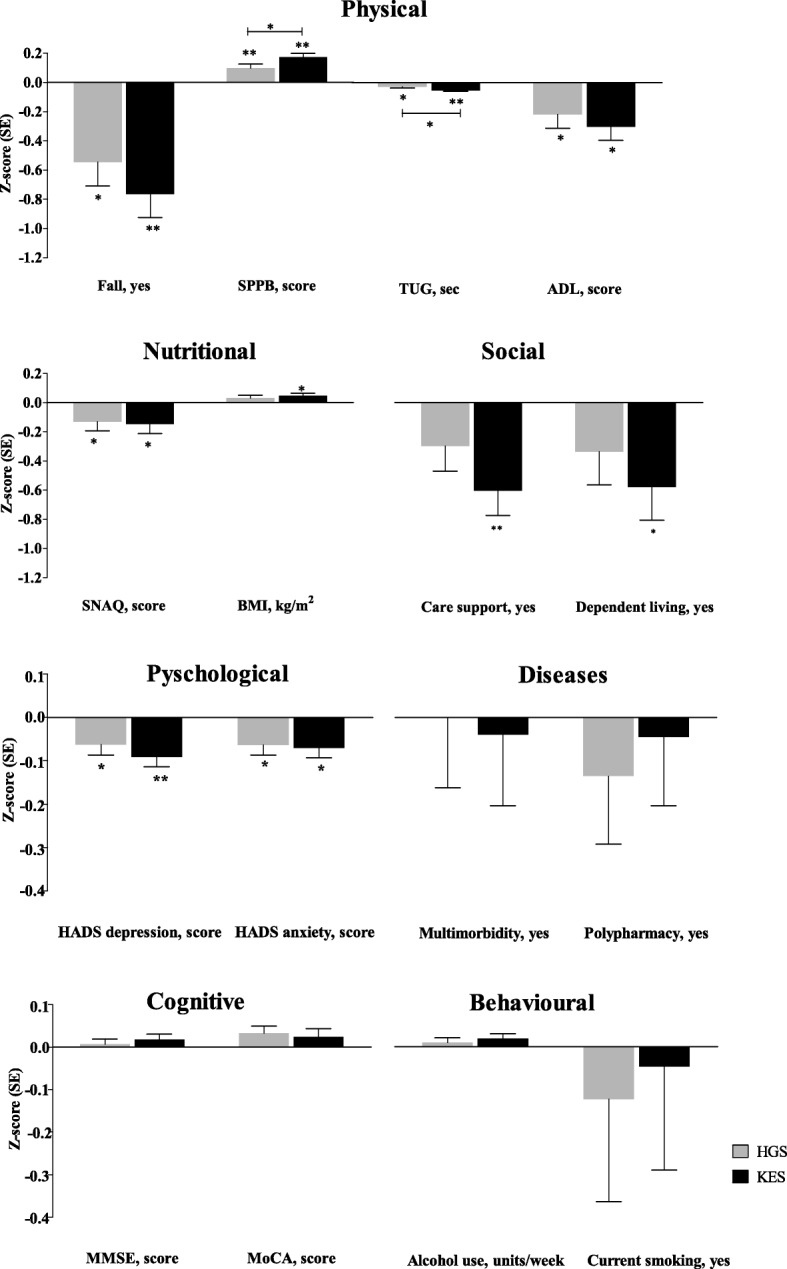
Associations between health characteristics and standardized handgrip strength and knee extension strength. *SE* standard error; *HGS* handgrip strength; *KES* knee extension strength; *SPPB* Short Physical Performance Battery; *TUG* Timed Up & Go; *ADL* Activities of Daily Living; *SNAQ* Short Nutritional Assessment Questionnaire; *BMI* body mass index; *HADS* Hospital Anxiety and Depression Scale; *MMSE* Mini-Mental State Examination; *MoCA* Montreal Cognitive Assessment. *P*-values were calculated with multivariate linear regression models adjusting for age and sex (model 1). HGS and KES are presented as sex-specific z-scores (SE). *p < 0.05, **p < 0.001

Table 2 shows the association between health characteristics with standardized HGS and KES. After adjustment for age and sex (model 1), physical (a fall in previous 12 months, lower SPPB score, slower in TUG test and higher ADL dependency), nutritional (higher SNAQ score), and psychological (higher HADS depression and anxiety score) characteristics were all significantly associated with lower HGS and lower KES respectively. Social (care support and dependent living) and nutritional (lower BMI) characteristics were significantly associated with lower KES but not with HGS. When comparing the effect estimates (β) of HGS and KES, the effect estimates of KES were significantly higher than the effect estimates of HGS for the SPPB (*p* = 0.038) and TUG test (p = 0.038). No significant differences in effect estimates of HGS and KES were found for other health characteristics. Figure 1 displays the associations between health characteristics with HGS and KES.

**Table 2:** The association between health characteristics with standardized handgrip strength (HGS) and knee extension strength (KES) in geriatric outpatients (*n* = 163)

	Model 1 (adjusted for age and sex)						Model 2 (Model 1+ Z KES)			Model 3 (Model 1+ Z HGS)		
	Z HGS			Z KES			Z HGS			Z KES		
	β	SE	p	β	SE	p	β	SE	p	β	SE	p
Physical												
Fall in previous 12 months, yes	− 0.546	0.164	**0.001**	− 0.764	0.160	**< 0.001**	− 0.168	0.154	0.275	− 0.506	0.145	**0.001**
SPPB, score	0.097*	0.027	**< 0.001**	0.173*	0.025	**< 0.001**	0.008	0.027	0.766	0.130	0.023	**< 0.001**
TUG, sec	−0.026*	0.009	**0.005**	− 0.051*	0.008	**< 0.001**	0.002	0.009	0.853	−0.039	0.008	**< 0.001**
ADL, score	−0.213	0.093	**0.023**	−0.298	0.090	**0.001**	−0.046	0.081	0.571	−0.185	0.078	**0.019**
Nutritional												
SNAQ, score	−0.131	0.063	**0.040**	−0.149	0.064	**0.020**	−0.054	0.055	0.329	−0.081	0.055	0.145
BMI, kg/m^2^	0.031	0.018	0.085	0.046	0.018	**0.012**	0.007	0.015	0.639	0.030	0.016	0.058
Social												
Care support, yes	−0.296	0.170	0.083	−0.600	0.167	**< 0.001**	0.023	0.151	0.879	−0.447	0.144	**0.002**
Dependent living, yes	−0.334	0.226	0.142	−0.574	0.226	**0.012**	−0.031	0.196	0.875	−0.397	0.194	**0.042**
Psychological												
HADS depression, score	−0.063	0.024	**0.009**	−0.091	0.023	**< 0.001**	−0.009	0.021	0.658	−0.056	0.019	**0.005**
HADS anxiety, score	−0.064	0.023	**0.006**	−0.070	0.023	**0.003**	−0.022	0.019	0.250	−0.032	0.019	0.097
Diseases												
Multimorbidity, yes	−0.001	0.162	0.994	−0.040	0.165	0.808	0.020	0.137	0.885	−0.039	0.139	0.778
Polypharmacy, yes	−0.136	0.158	0.390	−0.045	0.160	0.777	−0.112	0.134	0.404	0.027	0.136	0.842
Cognitive												
MMSE, score	0.007	0.012	0.560	0.018	0.012	0.124	−0.003	0.010	0.790	0.014	0.010	0.149
MoCA, score	0.032	0.017	0.065	0.024	0.019	0.207	0.021	0.015	0.166	0.006	0.016	0.730
Behavioural												
Alcohol use, units/week	0.010	0.011	0.352	0.019	0.011	0.080	0.000	0.009	0.994	0.014	0.009	0.139
Current smoking, yes	−0.122	0.238	0.609	−0.046	0.241	0.848	−0.096	0.197	0.626	0.023	0.200	0.907

*SPPB* Short Physical Performance Battery, *TUG* Timed Up & Go, *ADL* Activities of daily living, *SNAQ* Short Nutritional Assessment Questionnaire, *BMI* body mass index, *HADS* Hospital Anxiety and Depression Scale, *MMSE* Mini-Mental State Examination, *MoCA* Montreal Cognitive Assessment. In model 1, β of Z HGS and Z KES were compared with the formula ((β_1_- β_2_)/ √(SE_1_^2^-SE_2_^2^)) and *p* values derived from the standard normal distribution table and expressed as * if *p* < 0.05

Bold *p*-values represented significant association between health characteristics with standardized HGS or standardized KES based on linear regression

After further adjustment for standardized KES (model 2), the significant associations between health characteristics and HGS were lost. In contrast, after further adjustment for standardized HGS (model 3), the significant associations between health characteristics and KES remained, except for the association with SNAQ score, BMI and HADS anxiety score.

## Discussion

Health characteristics showed stronger associations with KES compared to HGS. All significant associations between health characteristics and HGS were lost after further adjustment for KES. Significant associations between health characteristics and KES (except for nutritional characteristics) were independent of HGS. The findings underpin the notion that measuring HGS alone may not be sufficient in clinical practice as a measure of muscle strength.

A limited number of studies examined the associations between health characteristics with both HGS and KES in the same individual [10–13]. Our findings are in line with previous studies which showed a stronger association between physical characteristics with KES compared to HGS. Among community-dwelling healthy older adults and institutionalized older adults, those with higher KES performed faster on TUG and no association between HGS with TUG was found [12, 13]. A stronger positive association between TUG with KES compared to HGS can be explained by the fact that performance on the TUG test largely depends on lower limbs muscles. In contrast, some studies in community-dwelling older adults demonstrated that physical performance was associated with both HGS and KES in the same individuals [10, 11]. The inconsistency in the association may be due to the use of six-minute walk test [10] and 12-m walk test [11] as physical assessments, in which other factor such as cardiovascular function plays a role in addition to muscle strength [23]. Additionally, KES and HGS were measured in only one leg and one hand [10, 11] instead of both sides of the limbs in the current study.

The association between health characteristics with HGS is dependent on KES, but the association between health characteristics with KES is independent of HGS. The change in effect sizes were larger when adjusting for KES compared to the model adjusting for HGS, indicating that KES showed a more confounding effect compared to HGS. An important factor in the superiority of KES over HGS in explaining variance in health characteristics in the present population may be the fact that the decline of muscle strength with aging is greater for the lower limb muscles than that of the upper limb [24]. Older adults are generally sedentary and use their lower limbs relatively less than their upper limbs for daily living activities [25]. The rate of KES loss as a function of age was found to be up to twice that of the loss of HGS (56% vs. 30%) in community-dwelling older adults [26]. Additionally, HGS was found to be not as sensitive as KES to environment [27, 28]. Substantial improvement in KES as high as 40% but without measurable changes in HGS was noted in whole-body resistance-type exercise training among frail older adults [27]. Even in study examining daily physical activity, significant positive association with KES but not HGS was found in community-dwelling older adults [28]. It has been shown that the agreement between HGS and KES was low, independently of age and health status [9], indicating that HGS or KES measurement cannot be used interchangeably. Previous reports underlie that HGS may be more suited for assessments of a state (of health) while the more change- sensitive KES may be used as a follow-up parameter [27, 28].

### Clinical implications

Whether clinicians should measure both HGS and KES in clinical practice is an important question to be answered because of their differences in terms of practicality in measurement and sensitivity to environment. Our findings underpin the notion that measuring HGS alone may be not sufficient in clinical practice and KES should be measured next to HGS. However, KES measurement in clinical practice is limited by the need for special equipment and training [7]. Instruments used to assess KES also varied and no validity data were found for some instruments [29]. Even with the same instrument to measure KES, the protocol and the cut-offs were not the same [30]. Currently, implementing KES measurement in clinical practice is hindered by the lack of data on valid protocols. Further effort is needed to reach a standardized procedure for measuring KES in geriatric clinical practice. In spite of the literature gap, based on the existing knowledge, clinicians are recommended to implement their standardized procedure and use instrumented KES measurement such as hand-held dynamometry [31] and isokinetic dynamometry [32] which have been shown to be reliable for measuring KES.

### Strengths and limitations

To the best of our knowledge, this is the first study comparing HGS with KES in their association with a variety of health characteristics in geriatric outpatients. A geriatric outpatient population is a unique population and highly relevant for clinical practice. This study also used objective measurements of physical performance in addition to self-reported functional limitation. A few limitations should be noted. First, this is a cross-sectional study so causal associations cannot be drawn. Second, our cohort is only composed of geriatric outpatients and therefore the results are limited to this specific population. Third, our analysis did not examine the effects of physical activity level, which is potentially important confounder for the association of interest.

## Conclusion

Health characteristics i.e. physical, nutritional and psychological characteristics are more associated with KES than HGS. The association between these characteristics with KES was independently of HGS. These findings indicate the additional value to include KES in the CGA.

## Abbreviations

ADL: Activities of daily living
BIA: Bioelectrical impedance analysis
BMI: Body mass index
CGA: Comprehensive Geriatric Assessment
HADS: Hospital Anxiety and Depression scale
HGS: Handgrip strength
IADL: Instrumental activities of daily living
IQR: Interquartile range
KES: Knee extension strength
MMSE: Mini-Mental State Examination
MoCA: Montreal Cognitive Assessment
SD: Standard deviation
SNAQ: Short Nutritional Assessment Questionnaire
SPPB: Short Physical Performance Battery
SPSS: Statistical Package for the Social Sciences
TUG: Timed Up and Go
